# The Role of Fatty Acid Binding Protein 3 in Cardiovascular Diseases

**DOI:** 10.3390/biomedicines10092283

**Published:** 2022-09-14

**Authors:** Ben Li, Muzammil H. Syed, Hamzah Khan, Krishna K. Singh, Mohammad Qadura

**Affiliations:** 1 Division of Vascular Surgery, St. Michael’s Hospital, Unity Health Toronto, University of Toronto, Toronto, ON M5B 1W8, Canada; 2 Department of Medical Biophysics, Schulich School of Medicine & Dentistry, Western University, London, ON N6A 5C1, Canada; 3 Department of Surgery, University of Toronto, Toronto, ON M5S 1A1, Canada; 4 Keenan Research Centre for Biomedical Science, Li Ka Shing Knowledge Institute, St. Michael’s Hospital, Unity Health Toronto, University of Toronto, Toronto, ON M5B 1W8, Canada

**Keywords:** fatty acid binding protein, FABP3, cardiovascular diseases, biomarker

## Abstract

Fatty acid binding proteins (FABPs) are proteins found in the cytosol that contribute to disorders related to the cardiovascular system, including atherosclerosis and metabolic syndrome. Functionally, FABPs serve as intracellular lipid chaperones, interacting with hydrophobic ligands and mediating their transportation to sites of lipid metabolism. To date, nine unique members of the FABP family (FABP 1–9) have been identified and classified according to the tissue in which they are most highly expressed. In the literature, FABP3 has been shown to be a promising clinical biomarker for coronary and peripheral artery disease. Given the rising incidence of cardiovascular disease and its associated morbidity/mortality, identifying biomarkers for early diagnosis and treatment is critical. In this review, we highlight key discoveries and recent studies on the role of FABP3 in cardiovascular disorders, with a particular focus on its clinical relevance as a biomarker for peripheral artery disease.

## 1. Introduction

Cardiovascular disease (CVD) is a major contributor to mortality rates globally, leading to ~17.5 million deaths annually [1]. Atherosclerosis, endothelial dysfunction, and dyslipidemia remain significant drivers of CVD [2]. Clinically, complications of blood vessels leading to coronary, cerebrovascular, and peripheral artery disease (PAD) account for most CVD-related morbidity and mortality [3].

PAD is a devastating CVD that involves lower extremity arterial atherosclerosis, affecting over 200,000,000 patients globally [4]. Although this condition significantly increases the risk of amputation and death, PAD remains poorly diagnosed and treated [5]. Compared to patients with coronary artery disease (CAD), PAD patients generally have an inferior prognosis over the long term [6]. One reason is the lack of a biomarker for PAD diagnosis and prognosis, as there is no PAD equivalent for cardiac troponin, a widely used biomarker in CAD [7]. Consequently, PAD patients are often misdiagnosed and receive delayed care [8].

Currently, the only accepted PAD screening tool is the ankle brachial index (ABI), but it has its fair share of limitations, including but not limited to unreliability in adults with diabetes, dependence on the test administrator, and challenging interpretability [9,10]. In a recent survey conducted by our group, we demonstrated that primary care physicians rarely perform ABI’s as they lack comfort with ordering and interpreting the test [11]. Additional barriers cited include time and staff constraints, limited equipment, and inadequate training [12]. Notably, studies suggest that physicians only correctly diagnose PAD patients about 50% of the time [13]. This results in delayed initiation of medical therapy, risk factor control, and surgical intervention, all of which increase a patient’s amputation and mortality risk [14]. Therefore, a clinically integrated, PAD-specific biomarker may aid healthcare practitioners in identifying high-risk patients in need of aggressive medical management, close follow-up, or surgery [7].

Fatty acid binding proteins (FABP) are intracellular proteins responsible for the transportation of lipids [15]. To date, researchers have identified nine unique isoforms of this protein that share the mechanism of interacting with lipid ligands and escorting them to metabolic sites [15]. The unique function of each isoform remains an area of heavy investigation [16]. Nevertheless, the role of FABPs in lipid processing within cells involved in atherosclerosis, including adipocytes, macrophages, and endothelial cells, suggests their importance in CVD development [17]. FABP3 has been shown to be associated with an assortment of CVDs.

One member of the FABP family, FABP3, is a small protein found intracellularly within cardiac or skeletal muscles that become released into circulation and excreted into the urinary system following injury to skeletal muscle, which may occur secondary to PAD-related tissue ischemia [18,19,20]. Previous work in this area has shown that FABP3 plays a role in metabolic syndrome, dysfunction of the mitochondria, and CVD [21,22]. In this review, we will highlight several recent studies demonstrating the usefulness of FABP3 as a potential biomarker for the diagnosis and prognosis of patients with PAD.

## 2. Cardiovascular Diseases

### 2.1. Epidemiology

A circulatory disorder, CVD primarily affects blood vessels in the heart, brain, and limbs [23]. Ischemic CVDs are common and involve stenoses in arteries supplying blood to critical organs [23]. This ultimately leads to clinical presentations such as myocardial infarction, stroke, and limb ischemia [23].

According to the World Health Organization, CVD contributes to 17,000,000 deaths in the world, and over 70% of mortality occurs in developing countries [24]. The devastating impact of CVD continues to increase and is anticipated to result in over 20,000,000 deaths by 2030 [24].

With regards to the economic burden, the estimated 2014 cost of CVD in the US was $351 billion, of which $213 billion were direct costs (e.g., medications and medical care) and the remaining $138 billion were indirect costs (e.g., loss of productivity, disability, and premature death) [25,26]. The burden of CVD shows no signs of slowing down, with CVD-related costs in 2035 forecast to reach over $1.1 trillion [25,26].

### 2.2. Risk Factors

Poor diets and lack of physical activity are major modifiable risk factors for CVD as they lead to high lipid levels in the blood [27]; this is a driving mechanism for atherosclerosis, a major contributor to CVD [27]. Furthermore, smoking increases the risk of CVD by causing damage to the endothelium, which is the innermost cell lining of blood vessels [28]. Non-modifiable risk factors for CVD include older age, male sex, and family history of CVD, hypercholesterolemia, and hypertension, among others [29,30]. In those who are older, CVD risk is often related to higher serum cholesterol levels and degeneration of the vascular system leading to loss of arterial elasticity [31].

### 2.3. Etiology

The basis of ischemic CVD is atherosclerosis, a chronic disorder characterized by the development of lipid-rich plaques on blood vessel walls leading to stenosis and turbulent flow [32]. The atherosclerotic process begins with the formation of fatty streaks, soft lesions containing lipid deposits and foam cells [32]. Over time, these entities protrude into the vascular lumen, restricting flow and causing downstream ischemia [32]. The development of fatty streaks is primarily driven by hypercholesterolemia, with other mechanisms playing a secondary role [33]. Circulating low-density lipoproteins (LDL) can cross the endothelium to reach the vessel’s intimal layer [33], where LDL is oxidized, triggering inflammation [33]. In response, circulating leukocytes become recruited and engage in the uptake of the oxidized LDL [33]. At this point, some of the leukocytes undergo apoptosis due to oxidative stress and become foam cells stuck to the injured vessel wall [33]. Over time, the foam cells form the plaque’s lipid core [33]. Oxidized LDL in the intima also stimulates vascular smooth muscle cells to migrate up to the subendothelial layer and proliferate, forming a fibrous structure around the lipid core [33]. As the lipid core and fibrous cover develop, the atherosclerotic plaque matures [33]. Over time, this plaque is susceptible to rupture and thrombosis, leading to complete blockage of the vessel lumen and significant downstream ischemia to critical organs [34]. Furthermore, in a process called embolization, pieces of the clot may break, circulate, and lodge in other vessels, causing acute ischemia and organ failure [34].

## 3. The Role of the Endothelium in CVD

The endothelium (inner wall of blood vessels) is directly in contact with blood and circulating biomolecules [35]. In the human body, there are approximately 35 trillion endothelial cells, occupying a surface area of >600 m^2^ [36]. The endothelium serves as a barrier between blood and the vascular matrix and acts as a mediator of gaseous/nutrient exchange between blood and tissues [36]. The endothelium also has important roles in regulating vascular tone, inflammation, and wound healing [37]. In addition, endothelial cells (ECs) facilitate angiogenesis and cellular migration, which are vital in congenital development, wound healing, and malignancy [37].

Normally, ECs do not promote thrombogenesis or inflammation [38]. Upon stressful stimulation, injured ECs are activated, becoming more permeable and contributing to inflammation, thrombosis, and vasoconstriction [38]. Hemodynamic or oxidative stresses, infectious pathogens, and metabolic insults such as hypoxia are common activators of ECs [39]. Exposure to stressful stimuli for long periods can result in endothelial dysfunction, which is characterized by inflammation, hypercoagulability, impaired barrier functions, dysregulated production of vasoactive factors, and enhanced mediators of hypertension [39].

Endothelial dysfunction is an important contributor to atherosclerosis and CVD development [40]. During the process of atherosclerosis, dysfunction of the endothelium is precipitated by oxidized LDL. This accelerates plaque formation and progression in 3 main ways: (1) reduced barrier function facilitates LDL penetration into the vessel wall, (2) increased nitric oxide production promotes thrombosis, and (3) injured ECs increase the production of inflammatory and coagulative molecules, facilitating leukocyte and platelet adhesion [41].

## 4. Fatty Acid Binding Proteins

FABPs are 12–15 kDa cytosolic proteins primarily found in tissues with high levels of lipid metabolism, such as the heart, skeletal muscle, and liver [42]. They are also found in cell types specialized for lipid storage, transportation, and signaling, such as macrophages and adipocytes [42]. FABPs have been demonstrated to be important in various metabolic and cardiovascular disorders, including atherosclerosis, obesity, and hyperglycemia [42]. All FABP members share a β-barrel signature containing a cavity filled with water and a lipid ligand binding site [42].

FABP 1–9 are nine members of the FABP3 family identified to date, each with 20–70% sequence homology [20]. Members are named based on the tissue that most abundantly expresses the protein [20]. As an example, FABP3 is also known as heart-type FABP as it is found primarily in cardiomyocytes, while FABP4/5 is expressed most prominently in adipocytes and epidermal cells [20].

Functionally, FABPs serve as lipid chaperones by reversibly interacting with hydrophobic ligands and mediating their escort to sites of lipid metabolism [43]. Target sites include the plasma membrane (lipid transport), mitochondria (lipid metabolism), and endoplasmic reticulum (lipid biosynthesis) [43]. Although lipid signaling is related to FABP expression, the specific function of each member of the FABP family is poorly understood [15]. This is due to the fact that their mechanisms relate to many complex regulatory pathways [15].

## 5. FABP3

FABP3 is most abundantly expressed in skeletal and heart muscle [44]. As a chaperone for lipids, FABP3 is critical for maintaining the homeostatic function of skeletal and cardiac muscle [44]. Seventy percent of muscle cell energy is derived from fatty acid oxidation in mitochondria, which requires lipid transportation [45]. Studies of FABP3-deficient mice show compromised uptake of fatty acids, reduced capacity for exercise, and rapid glucose consumption, ultimately leading to cardiac dysfunction [46].

FABP3 has been demonstrated to be a biomarker for heart injury, particularly myocardial infarction (MI) [47]. During cardiomyocyte injury, cellular components are broken down and released into the circulation [48]. Myocardial protein leakage serves as a biomarker of pathology that can be measured to allow early detection of cardiac injury [49]. Currently, troponin is a widely used biomarker for MI, which becomes elevated 2–4 h after the development of chest pain [50]. In contrast, serum FABP3 levels begin to rise 30 min after chest pain onset and peak in a few hours [51]. Early FABP3 release from injured myocardium has been demonstrated in animal models and humans [52,53]. Therefore, FABP3 has the potential to be an important biomarker for coronary artery disease [54]. Of recent interest, FABP3 has been linked to atherosclerosis in other blood vessels owing to its contribution to endothelial dysfunction [55].

Recently, our research group has conducted several studies demonstrating the utility of FABP3 as a biomarker for PAD [56,57,58]. PAD patients essentially have an acquired myopathy secondary to repetitive tissue ischemia and reperfusion [59]. This ultimately leads to abnormal changes in mitochondrial enzyme expression, increased oxidative stress, and apoptosis [59]. This process can contribute to the release of FABP3 into the circulation and urine. Herein, we summarize our findings.

## 6. Association between Serum FABP3 and PAD

Previously, we assessed the differential expression and association between serum FABP3 levels and diagnosis of PAD among a cohort of 75 patients with PAD and 75 non-PAD patients [56]. Our data demonstrated that patients with PAD had significantly higher blood levels of FABP3 than those without PAD, even after accounting for confounding factors. Furthermore, FABP3 levels were shown to have a directly proportional relationship with PAD severity—the more advanced the PAD, the higher the FABP3 levels. These findings were also validated through muscle biopsy results demonstrating higher levels of FABP3 expression in patients with PAD compared to those without PAD.

Based on these data, serum FABP3 may be a valuable diagnostic biomarker that aids first-line physicians and healthcare practitioners with the timely screening of patients for PAD, assessing the severity and extent of the disease, and guiding decision-making regarding medical therapy. If integrated correctly within clinical practice, a FABP3-based diagnostic test for PAD has immense potential in lowering the morbidity and mortality currently associated with the disease.

## 7. Association between Urinary FABP3 and PAD

Since FABP3 is filtered by the kidneys and excreted into the urine, we further investigated whether urinary FABP3 is associated with PAD as well as its inherent potential to be a diagnostic biomarker for PAD [57]. Based on data from 130 patients (65 with PAD and 65 without PAD), we noted 1.7 times increased urinary FABP3 levels in the PAD group than in the non-PAD group. This association between urinary FABP3 and PAD remained robust even after adjusting for confounders. Furthermore, a directly proportional relationship between increasing urinary FABP3 levels and PAD severity was observed, and ROC analysis suggested that urinary FABP3 levels have a good discriminatory ability for distinguishing PAD from non-PAD cases. Overall, this body of work lays the fundamental groundwork for future studies investigating FABP3 as a PAD biomarker. Supplementing our serum FABP3 study, urinary FABP3 may also be a novel and important diagnostic biomarker for PAD. Given that urine tests are less invasive than blood tests, widespread clinical adoption and uptake may be easier.

## 8. Pathophysiological Underpinnings of the Relationship between FABP3 Levels and PAD

While the exact pathophysiology delineating the relationship between FABP3 levels and PAD is yet to be understood, plausible insights, explanations, and hypotheses could be generated from reviewing the literature.

**Figure 1:**
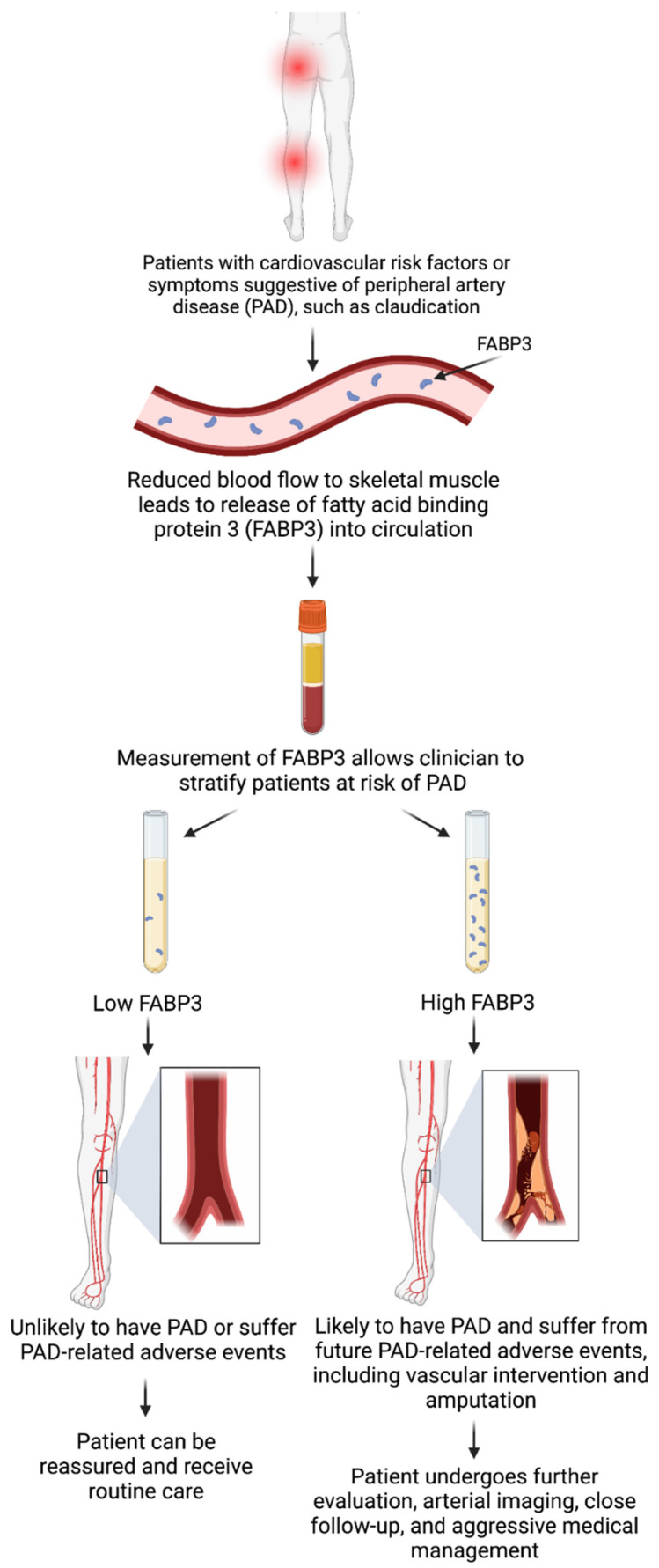
Clinical workflow for the use of fatty acid binding protein 3 (FABP3) as a diagnostic and prognostic biomarker for peripheral artery disease (PAD). Created using BioRender with permission.

Patients experiencing myopathies have been shown to release FABP3 after incurring injury to the skeletal muscle (exercise-induced or otherwise) [60]. PAD can be considered a form of myopathy following skeletal muscle injury, primarily due to tissue ischemia and reperfusion [61]. These often translate to detrimental changes in mitochondrial oxidative stress, enzymes, and respiration, ultimately leading to muscle apoptosis [59]. As a byproduct of these cellular processes and changes, FABP3 is potentially released into the circulation [62]. Furthermore, the extent of skeletal muscle injury could result in more/less release of FABP3 into circulation—potentially explaining our observed finding of a relationship between increased PAD severity and elevated FABP3 levels [18]. This could be a plausible mechanism explaining the pathophysiology of our findings but requires further investigation and confirmation. Figure 1 highlights the clinical workflow for the potential use of FABP3 as a diagnostic and prognostic biomarker for PAD.

## 9. Clinical Studies of FABP3 as a Biomarker for Various Disease States

A growing body of literature suggests that FABP3 may be used as a biomarker for other disease states. For instance, Tanaka and colleagues showed an elevation of uFABP3 in patients with MI [63]. Other groups, including Nayashida et al., studied the impact of kidney function on uFABP3 levels in patients undergoing coronary artery bypass grafting and showed that uFABP3 was a marker for myocardial injury [64]. The literature suggests that following injury to the myocardium, kidney filtration of FABP3 can be impaired from acute kidney injury due to reduced creatinine clearance, resulting in lower levels of uFABP3 [65]. Therefore, urinary FABPs may also help in expediting the diagnosis of acute renal failure [66,67]. Most recently, our group demonstrated that uFABP3 is associated not only with PAD diagnosis but major adverse limb events related to PAD [58]. This demonstrates that uFABP3 has both diagnostic and prognostic value in identifying PAD patients with a high risk of complications and who may require more intensive medical and surgical therapy [58].

## 10. Conclusions

In this review, we highlighted the epidemiology and pathophysiology of CVD, with a focus on peripheral artery disease and its important association with mortality and limb loss. We described the roles of FABPs in cardiovascular disease and specifically highlighted FABP3′s potential utility as a biomarker for PAD. We summarized the findings of recent translational studies from our group demonstrating both the diagnostic and prognostic value of serum and urinary FABP3 in PAD. Overall, our review highlights the promising role of FABP3 in early diagnosis and risk stratification of high-risk individuals for further evaluation, close follow-up, and aggressive management to reduce the significant morbidity and mortality associated with cardiovascular disorders.

## Data Availability

All relevant data is reported in the article. Additional data is available upon request from the corresponding author.
